# Acknowledgement to Reviewers 2023

**DOI:** 10.3389/phrs.2024.1607101

**Published:** 2024-01-31

**Authors:** PHR Editorial Office

**Affiliations:** Swiss School of Public Health (SSPH+), Zürich, Switzerland

The Editors of the *Public Health Reviews* would like to thank all Reviewers in 2023 for the time they have spent and the valuable contributions they have made to ensure the scientific quality of the journal.

Thomas Agyarko-Poku, Ghana

Latasha Allen, United States

Diogo Almeida, Portugal

Baye Amlak, Ethiopia

Daniela Anker, Switzerland

Saeed Anwar, Canada

Leonardo Araujo, Brazil

Nicolas Arriagada, Australia

Alex Backer, United States

Heather Bailey, United Kingdom

Helen Banks, Italy

Tess Bardy, Switzerland

Anke Berger, Switzerland

Sutanu Bhattacharya, United States

Aikant Bhatti, India

Mark Brittan, United States

Diana Buitrago-Garcia, Switzerland

Barbara Bürkin, Switzerland

Maribel Carvalhais, Portugal

Lorys Castelli, Italy

Luis Cereijo Tejedor, Spain

Joana Costa, Switzerland

Ana Cruz, Portugal

Katarzyna Czabanowska, Netherlands

Andrew Dannenberg, United States

Kathryn Dawson-Townsend, Switzerland

Silvia Deandrea, Italy

Sarah Dow-Fleisner, Canada

Abbas Ebrahimi-Kalan, Iran

Ugochukwu Eze, Nigeria

Marta Fadda, Switzerland

Filipa Fontes, Portugal

Lebo Gafane-Matemane, South Africa

Juan García-Iglesias, Spain

Nicole Geovana, Brazil

Olga Gershuni, Netherlands

Scott Greer, United States

Bern Grush, Canada

Ben Harris-Roxas, Australia

Herwansyah Herwansyah, Netherlands

Yao-Ching Huang, Taiwan

Devraj JP, India

Mehmet Kurutkan, Türkiye

Yu Lan, United States

Roberto Leombruni, Italy

Barry Levy, United States

Florian Liberatore, Switzerland

Cosima Lisi, Portugal

Erand Llanaj, Germany

Ramon Llopis-Goig, Spain

Dan Lupu, Romania

Olusesan Makinde

Sarah Mantwill, Switzerland

Gregory MD, United States

Niklaus Meier, Switzerland

Yayehirad Melsew, Australia

Matthias Merkel, United States

Bénédicte Mittaine-Marzac, France

Binyam Minuye, Ethiopia

Masoud Mohammadnezhad, United Kingdom

Ana Monteiro, Portugal

Sarah Murphy, Portugal

Jesús Rivera Navarro, Spain

Kagiso Ndlovu, Botswana

Anton Ninkov, Canada

Konrad Obermann, Germany

Osondu Ogbuoji, United States

Eva Pagano, Italy

Unmesha Paladhi, United States

Brandon Patterson, United States

Pamela Ransom, United States

Yasmin Rashid, Pakistan

Mayte Restrepo, United States

Mónica Rodrigues, Portugal

Pablo Rodríguez-Feria, Netherlands

Andres Roman-Urrestarazu, United Kingdom

Thomas Rotter, Canada

Olivier Rubin, Denmark

João Rufo, Portugal

Cláudia Santos, Switzerland

Leon Sawh, United States

Angela Schnelli, Switzerland

Laure-Elise Seghers, United Kingdom

Justin Sempsrott, United States

Giulia Sesa, Belgium

Shirin Sighaldeh, Iran

José Silva, Portugal

Ivica Smokovski, North Macedonia

Sara Soares, Portugal

Elena Strippoli, Italy

Sadeka Tamanna, United States

Lauren Terzis, United States

Joyce Thompson, United States

Carole Upshur, United States

Ezra Valido, Switzerland

Roberto Valiente, United Kingdom

Siquan Wang, United States

Marcia Weaver, United States

Jeremy Youde, United States

Yi Chien Yu, Taiwan

Renee Zahnow, Australia

Dong Zhang, United States

Kamila Zvolská, Czechia

